# The value of applied science

**DOI:** 10.1038/s41467-023-36307-4

**Published:** 2023-02-03

## Abstract

Every area of science can contribute to the changes that are required for a sustainable future through the application of its fundamental discoveries. While some fields have clear paths to application, *Nature Communications* believes that there is great potential for utility and application to be found in, and across, all of  the different disciplines that we publish.

In 1932, *Nature* published an article entitled Relations between Pure and Applied Science that argued strongly for the application of scientific ideas to get the recognition it deserved. In the intervening 90 years, the central thesis of the article still rings true: the importance of applied science should not be overlooked.

In medicine, there’s the often used term ‘bench to bedside’ to describe how fundamental discoveries of important biological insight become the techniques and medicines that save or improve lives. Similarly, for the development of new and improved materials, energy systems, and electronics, to name but a few, we need applied science to develop our initial discoveries into usable technologies. It is this application of insight into a system that brings discoveries out of the laboratory and into the everyday world. The line between the pure and the applied sciences is not a hard border, but a bridge linking the two.

Today’s world faces problems with complex and multifaceted causes. From the continuing impact of SARS-CoV-2 to the crisis of climate change and the work necessary to achieve the Sustainable Development Goals, science and technology will play an important role in addressing the problems we collectively face. If the world is to build a sustainable and equitable future, with rising standards of living for all, advances in our scientific understanding need to go from the bench to not just the bedside but into every aspect of life.

To provide a home for these studies, we recognise that manuscripts presenting applications should be assessed on different criteria from those reporting a novel discovery. It is important to sometimes put aside the requirement for novel insight and rather consider potential uses and current challenges faceing effective application. When reaching a decision on the suitability of such manuscripts for publication, we will use as guidance parameters such as the Technology Readiness Level [Box 1]—an estimation of the maturity of a technology originally developed by NASA and now widely adopted—of an application, as well as an assessment of how market ready a technology or application is.

“It is important to sometimes put aside the requirement for novel insight and rather consider potential uses and current challenges facing effective application.”

## Box 1

Technology readiness levels (TRL).

- TRL 1: Basic principles are observed and reported
- TRL 2: Concept or application is formulated
- TRL 3: Demonstration of analytical and experimental critical function or characteristic proof-of-concept
- TRL 4: Demonstration of basic validation of the technology in a laboratory environment
- TRL 5: Demonstration of basic validation of the technology in a relevant environment
- TRL 6: Model or prototype demonstration of the technology in a relevant environment
- TRL 7: Prototype demonstration of the technology in an operational environment
- TRL 8: Actual technology completed and qualified through testing and demonstration
- TRL 9: Actual technology qualified through successful operational use.

Just as editors and reviewers should think in terms of utility over discovery for those applied studies, we also encourage authors to consider the potential applications of their work and plan ahead for commercialisation and patentability when applicable. While as a journal we neither encourage nor discourage this—as only the authors of a work can make these decisions—the potential commercialisation of research should not be seen as a barrier to publication.

Our Perspectives and Comments have always provided a way for authors to give their views on where the research community should be heading. We recognise that the deployment of the applied sciences requires more than retrospective commissioned content reviewing a field’s development, and needs to include forward-facing papers that help to guide a field.

To help bridge the academic-commercial divide, we have reached out to authors that move in both worlds for insight and guidance on how to transition research from academia to industry. We have a Comment from William Hait and Paulus Stoffels at Johnson & Johnson, giving guidance on academic–industrial partnerships, and a discussion about commercialisation of research from an academic standpoint by Professor Kylie Vincent. Also, in the Q&A with Dr. Andrea Crottini we look at research commercialisation from the point of view of a technology transfer officer. To highlight some of the great content we receive in this area, our editors have also curated a Collection of research articles on applied science across the chemical, physical, materials, and biological sciences. We will continue to update this collection as new work is published, so be sure to check back regularly for the latest additions. We encourage you to submit your applied work to *Nature Communications* for consideration in the collection.

Whether your work involves fundamental basic science discoveries or the development of new technologies and methods, whether you wish to patent or not, whether the work is from an academic laboratory, an established company or a young start-up just spun out from a university, the editors at *Nature Communications* want to ensure we can support all of our authors in finding a home for their work.

